# miRNA-221 is elevated in cystic fibrosis airway epithelial cells and regulates expression of ATF6

**DOI:** 10.1186/s40348-014-0012-0

**Published:** 2015-01-07

**Authors:** Irene K Oglesby, Raman Agrawal, Marcus A Mall, Noel G McElvaney, Catherine M Greene

**Affiliations:** 1 Respiratory Research Division, Department of Medicine, Royal College of Surgeons in Ireland, Education and Research Centre, Beaumont Hospital, Dublin, 9 Ireland; 2 Department of Translational Pulmonology, Translational Lung Research Center, Member of the German Center for Lung Research, University of Heidelberg, 69120 Heidelberg, Germany; 3 Division of Pediatric Pulmonology & Allergy and Cystic Fibrosis Center, Department of Pediatrics, University of Heidelberg, 69120 Heidelberg, Germany

**Keywords:** ATF6, Cystic fibrosis, miRNA-221

## Abstract

**Background:**

MicroRNA (miRNA) and messenger RNA (mRNA) expression differs in cystic fibrosis (CF) versus non-CF bronchial epithelium. Here, the role of miRNA in basal regulation of the transcription factor ATF6 was investigated in bronchial epithelial cells *in vitro* and *in vivo*.

**Methods:**

Using *in silico* analysis, miRNAs predicted to target the 3′untranslated region (3′UTR) of the human ATF6 mRNA were identified.

**Results:**

Three of these miRNAs, miR-145, miR-221 and miR-494, were upregulated in F508del-CFTR homozygous CFBE41o- versus non-CF 16HBE14o- bronchial epithelial cells and also in F508del-CFTR homozygous or heterozygous CF (*n* = 8) versus non-CF (*n* = 9) bronchial brushings. ATF6 was experimentally validated as a molecular target of these miRNAs through the use of a luciferase reporter vector containing the full-length 3′UTR of ATF6. Expression of ATF6 was observed to be decreased in CF both *in vivo* and *in vitro*. miR-221 was also predicted to regulate murine ATF6, and its expression was significantly increased in native airway tissues of 6-week-old βENaC-overexpressing transgenic mice with CF-like lung disease versus wild-type littermates.

**Conclusions:**

These results implicate miR-145, miR-221 and miR-494 in the regulation of ATF6 in CF bronchial epithelium, with miR-221 demonstrating structural and functional conservation between humans and mice. The altered miRNA expression evident in CF bronchial epithelial cells can affect expression of transcriptional regulators such as ATF6.

## Background

Cystic fibrosis (CF) is a common autosomal recessive inherited disease. People with CF (PWCF) have a lower than normal average life expectancy [1]. Affected individuals typically become symptomatic in infancy and childhood and classically present with mucosal infections, exocrine pancreatic insufficiency and elevated sweat chloride levels. Although it is a multisystem disorder, recurrent lung infections are responsible for the major morbidity and mortality in PWCF.

CF occurs due to mutations in the gene encoding the CF transmembrane regulator (CFTR) protein, a cyclic AMP-activated chloride channel that controls the secretion of chloride and bicarbonate ions across the airways and other epithelial surfaces. Six classes of CFTR mutations have been described, with the class II F508del-CFTR mutation representing the most common CFTR mutation worldwide [2]. The misfolded F508del-CFTR protein encoded by this mutation accumulates in the endoplasmic reticulum (ER) and fails to reach the apical surface of epithelial cells to function as an anion channel. The βENaC-transgenic mouse phenocopies increased airway Na^+^ absorption and airway surface liquid depletion characteristic of human CF airways and develops spontaneous CF-like lung disease with mucus plugging, chronic airway inflammation and reduced clearance of bacterial pathogens [3,4].

The ER is the site of protein translation, folding and processing for transport to secretory vesicles. Perturbation of the ER can lead to a phenomenon termed ER stress which results in the initiation of signalling networks aimed at restoration of ER equilibrium. One ER stress network is the unfolded protein response (UPR). Activating transcription factor 6 (ATF6) is an ER resident transcription factor and a key component of the UPR [5]. Its activation leads to transcriptional induction of ATF6-regulated genes which function primarily to restore correct protein folding in the ER.

Recent evidence implicates microRNAs (miRNAs) in regulation of the UPR [6-9]. miRNAs are short regulatory RNAs that hybridise to miRNA recognition elements (MREs) located largely in the 3′UTR of mRNA transcripts, leading to mRNA degradation and/or inhibition of translation. Dysregulation of miRNA expression has been described in numerous disease states including CF, where the miRNA expression profile of bronchial epithelial cells has been shown to be altered [10].

To date, there have been no studies examining whether altered miRNA expression regulates expression of UPR genes in CF airway epithelium. Therefore, we undertook a study to examine miRNA expression and regulation of ATF6 in CF and non-CF airway epithelial cells *in vitro* and *in vivo*, and complemented these human studies by analysing the expression of key miRNAs in a mouse model of CF lung disease.

## Methods

### Study populations, bronchial brush sampling and miRNA profiling

Following informed consent under a protocol approved by the Beaumont Hospital ethics review board, bronchial brushings were sampled and RNA isolated as previously described [10] from 17 individuals: CF (*n* = 8, 27.2 + 2.7 years, M/F 5:3), confirmed by sweat testing and/or genotyping, (Table 1) and non-CF controls (*n* = 9, 50.8 + 5.4 years, M/F 5:4) (Table 2).

**Table 1:** **CF patient demographics**

**CF**	**Sex**	**Age**	**CFTR genotype**	**FEV**_**1**_	**BMI**	**PI**	***Ps*** **.**	***Sa*** **.**	**Asp.**
1	M	21	ΔF508/R506T > K	17%	30.5	Y	+	+	−
2	M	29	ΔF508/ΔF508	64%	20.24	Y	+	−	−
3	F	25	ΔF508/R177H 5T	89%	23.15	N	-	+	=
4	M	18	ΔF508/ΔF508	87%	20.89	Y	+	+	+
5	M	23	ΔF508/unknown	27%	17.76	Y	+	-	-
6	F	19	ΔF508/ΔF508	54%	21.71	Y	+	-	-
7	F	25	ΔF508/1717GA	51%	19.62	Y	+	-	+
8	M	31	ΔF508/621 + 1G → T	83%	22.77	Y	+	-	+

FEV_1_, forced expiratory volume in 1 s percent predicted; BMI, body mass index; PI, pancreatic insufficiency (Y, yes; N, no); Ps., colonisation with *Pseudomonas aeruginosa*; Sa., colonisation with *Staphylococcus aureus*; Asp., colonisation with *Aspergillus* species.

**Table 2:** **Non**-**CF control patient demographics**

**Non**-**CF**	**Sex**	**Age**
1	M	47
2	M	57
3	F	68
4	F	73
5	F	60
6	M	54
7	F	20
8	M	47
9	M	46

All animal studies were approved by the Animal Care and Use Committee of the Regierungspräsidium Karlsruhe, Germany.

### Cell lines and culture conditions

Human bronchial epithelial 16HBE14o- and F508del homozygous CFBE41o- cell lines were obtained as a gift from D. Gruenert (California Pacific Medical Centre Research Institute, San Francisco, CA, USA). HEK293 (human embryonic kidney cell line) were obtained from the European Collection of Cell Cultures (Salisbury, Wiltshire, UK). Cells were routinely grown in minimum essential media (MEM + GlutaMax, Gibco, Life Technologies, Carlsbad, CA, USA) supplemented with 10% fetal calf serum (Gibco) and 1% penicillin-streptomycin (Gibco) in 75-cm^2^ flasks and maintained in a 37°C humidified incubator containing 5% CO_2_. For experiments, cells were sub-cultured once 60% to 80% confluency was achieved and seeded at optimised densities.

*In silico analysis*. Bioinformatic analysis was performed using the miRNA target prediction database TargetScan 6.2 to search for putative targets of miRNA differentially expressed in CF airway epithelium. Subsequently, a range of databases (including miRWALK http://www.umm.uni-heidelberg.de/apps/zmf/mirwalk/micrornapredictedtarget.html which incorporates MicroRNA.org, PITA, miRDB and Microcosm) were interrogated to search for miRNA targeting human and murine ATF6.

### Measurement of human miRNA expression levels by qRT-PCR

Total RNA was extracted using TriReagent (Sigma-Aldrich, St. Louis, MO, USA). Generation of cDNA for miRNAs of interest was performed using Taqman MicroRNA Reverse Transcription kits (Applied Biosystems, Foster City, CA, USA). miRNA expression in brushings and cell lines was measured on a Roche LC480 Lightcycler (Roche, Penzberg, Bavaria, Germany). Expression of miRNAs relative to miR-218 was determined using the 2^(−ΔΔCt)^ method. miR-218 was chosen for normalisation due to a high degree of similarity in expression levels between CF and non-CF brushings observed in the expression profiling screen. All quantitative real-time polymerase chain reaction (qRT-PCR) experiments in cell lines were performed in triplicate, a minimum of three times and included no-template controls.

### Mouse studies

The βENaC-transgenic mouse was originally generated on a mixed genetic background (C3H/He × C57BL/6) and was backcrossed to the C57BL/6 background as previously described [11]. Experimental animals were housed in a specific pathogen-free animal facility of the University of Heidelberg and had free access to chow and water. Transgene-positive mice were identified by PCR of genomic DNA [3], and wild-type (WT) littermates served as controls. The trachea and main stem bronchi were collected from 6-week-old mice as previously described [12], and total RNA was isolated using TRIzol reagents (Invitrogen, Life Technologies, Carlsbad, CA, USA). Quantitative real-time PCR for miRNAs was performed with TaqMan assays (Applied Biosystems, Foster City, CA, USA) as per the manufacturer's protocols.

### Luciferase reporter plasmid transfection

HEK293 cells (1 × 10^5^ in triplicate) were transiently co-transfected for 24 h with a WT-ATF6 3′UTR (OriGene, Rockville, MD, USA) firefly luciferase reporter vector containing the full-length 3′UTR (250 ng), a constitutive *Renilla* luciferase vector (100 ng) and 30 nM synthetic premiR mimics (PM) for miR-145, miR-221 and miR-494 (Applied Biosystems, Foster City, CA, USA) as indicated or with a scrambled control. PremiR-223, a miRNA increased in the CF lung but not predicted to target ATF6, was included as a control. Transfections were performed using Genejuice (Novagen, Madison, WI, USA) for plasmid DNA and Ribojuice (Novagen, Madison, WI, USA) for miRNA in OptiMEM reduced serum media (Life Technologies, Carlsbad, CA, USA) as per the recommended conditions. Lysates were prepared and measurement of luciferase was performed using the luciferase assay system (Promega, Madison, WI, USA). Relative luciferase activity was calculated. Endogenous red fluorescent protein (RFP) expressed by the ATF6 3′UTR reporter was used to monitor transfection efficiency.

### Measurement of mRNA expression levels by qRT-PCR

Total RNA was extracted using TriReagent. For mRNA gene expression, equal quantities of RNA were reverse transcribed into cDNA using the Quantitect Reverse Transcription Kit (Qiagen, Valencia, CA, USA) following the manufacturer's protocol. Expression of ATF6 mRNA relative to *β-actin* (data presented as fold differences) was determined using the 2^(−ΔΔCt)^ method (details on primers are shown in Table 3).

**Table 3:** **Primers used in this study**

**Gene**	**Primers** **(5′** **-3′)**	**Bases spanned**	**Product size** **(bp)**
hATF6^a^	F-TGAACTTCGAGGATGGGTTC	1,560 to 1,739	180
	R-TCACTCCCTGAGTTCCTGCT		
hβ-actin	F-GGACTTCGAGCAAGAGATGG	747 to 884	138
	R-AGGAAGGAAGGCTGGAAGAG		

^a^h, human.

### Statistical analysis

All analyses were performed using GraphPad PRISM 4.0 (GraphPad Software Inc., San Diego, CA, USA). Results are expressed as the mean ± SEM and were compared by Student's *t* test. Differences were considered significant at *p* ≤ 0.05.

## Results

### ATF6 is predicted to be regulated by miRNAs that are upregulated in CF airway epithelium

**Figure 1:**
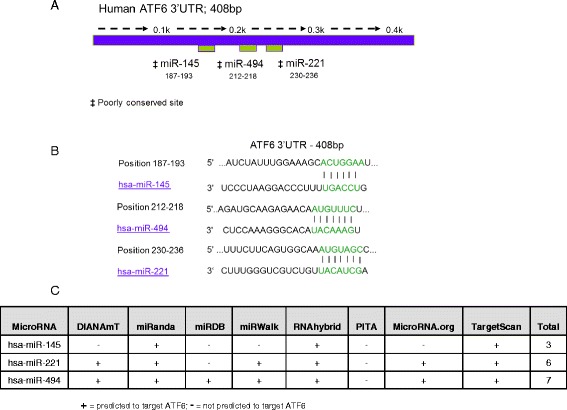
**miR-** **145,** **miR-** **221 and miR-** **494 are predicted to target human ATF6. (A)** Predicted binding location of miR-145, miR-221 and miR-494 in the full-length (408 bp) human ATF6 3′UTR and **(B)** proposed base pair matches for miR-145, miR-221 and miR-494 within the 3′UTR of ATF6 as predicted by TargetScan 6.2. **(C)** Predicted targeting of the human ATF6 3′UTR by miR-145, miR-221 and miR-494 by DIANAmt, miRanda, miRDB, miRWALK, RNAhybrid, PITA, MicroRNA.org and Targetscan 6.2.

We previously reported differential expression of 68 miRNAs in CF versus non-CF bronchial brushings by *in situ* qRT-PCR [10]. *In silico* analysis of potential targets of these differentially expressed miRNAs was performed. ATF6, a protein of interest to us, was predicted to be regulated by three of the upregulated miRNAs - miR-145, miR-221 and miR-494 (Figure 1). Figure 1A depicts the full-length human ATF6 3′UTR with predicted binding locations for miR-145, miR-221 and miR-494, and Figure 1B shows the locations and base pair matches of their proposed binding sites, adapted from TargetScan 6.2. A range of other target prediction databases were interrogated, and ATF6 was listed as a putative target of two or more of these miRNAs in six of the eight databases interrogated, Figure 1C, each with only one miRNA recognition element predicted in the ATF6 3′UTR.

### miR-221 is increased in CF versus non-CF cell lines and bronchial brushings

**Figure 2:**
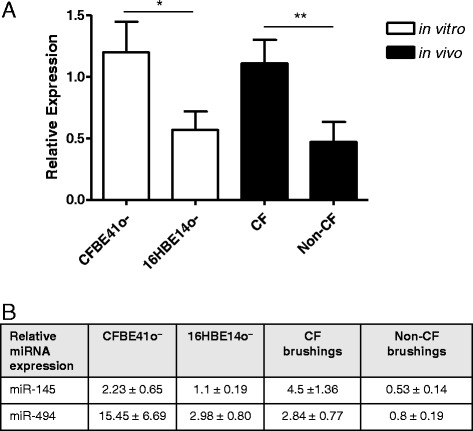
**Relative expression levels of miR**-**221 in CF versus non**-**CF bronchial epithelial cell lines and bronchial brushings. (A)** Relative expression of miR-221 was determined by qRT-PCR using individual TaqMan human miRNA assays and normalised to miR-218 in the CF bronchial epithelial cell line CFBE41o- compared with its non-CF counterpart 16HBE14o- (three separate cultures measured in triplicate) and in bronchial brushings (CF, *n* = 8; non-CF, *n* = 9). Data are represented as mean ± SEM and were compared by *t* test (*p < 0.05, **p < 0.01). All qRT-PCR experiments for cell lines were performed in triplicate and included no-template controls. **(B)** Relative expression of miR-145 and miR-494 normalised to β-actin in the CF versus non-CF cell lines and bronchial brushings used in (A).

Figure 2A shows that miR-221, which was significantly elevated with a median fold change of 1.92 in CF versus non-CF bronchial brushings as shown by miRNA profiling [10], is similarly increased in CF versus non-CF cell lines (*p* < 0.05) and also in CF versus non-CF bronchial brushings (*p* < 0.01).

We have previously reported that miR-145 and miR-494 are increased *in vitro* and *in vivo* in CF versus non-CF bronchial epithelium [13]. Figure 2B shows the levels of these miRNAs, normalised to miR-218, in the samples studied here.

### Expression of miR-145, miR-221 and miR-494 is increased in airway tissues from βENaC-transgenic versus wild-type mice

**Figure 3:**
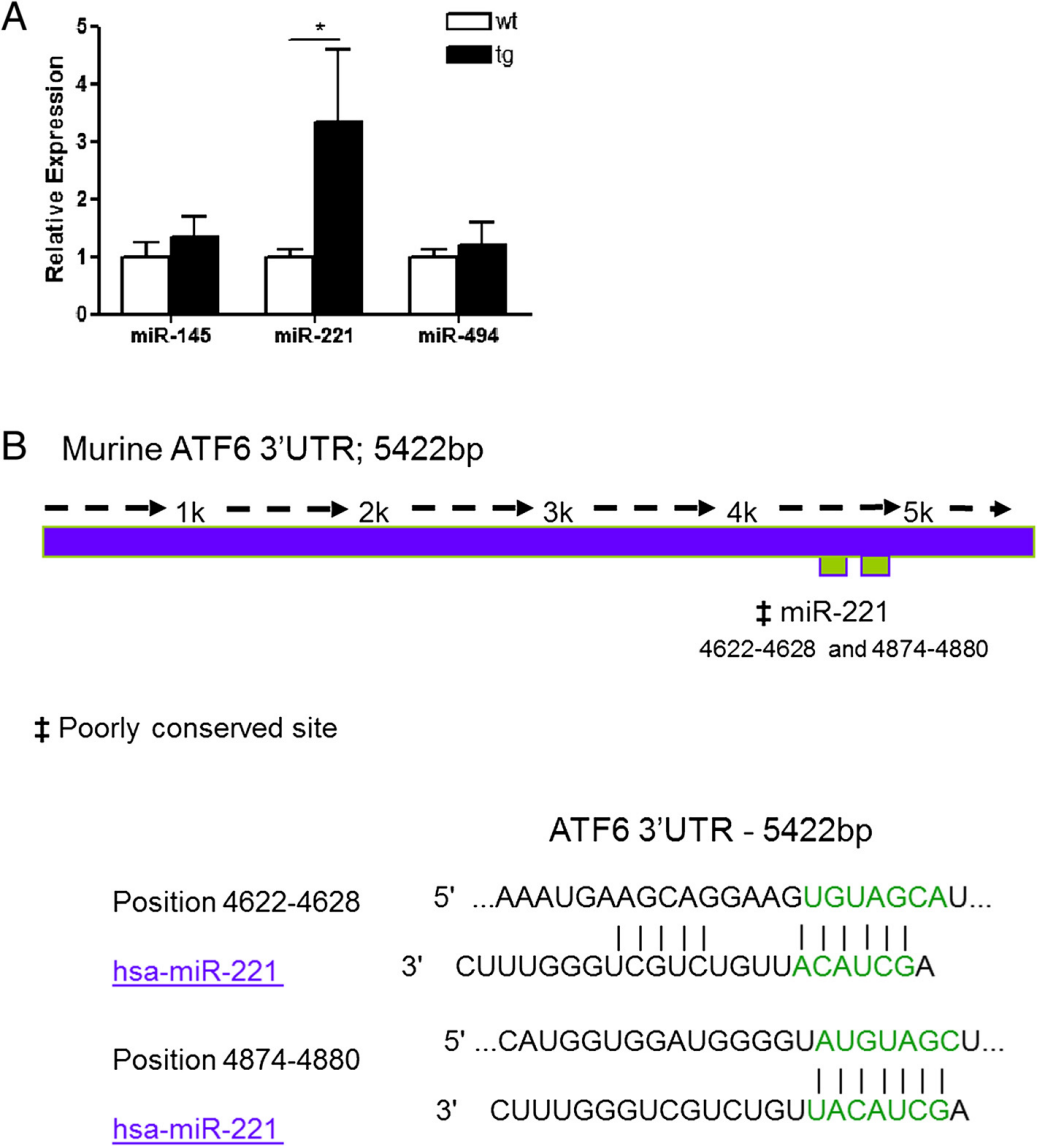
**miR-221 is increased in trachea and bronchi and is predicted to target murine ATF6 3’UTR. (A)** Relative expression of murine miR-145, miR-221 and miR-494 was determined by qRT-PCR using individual TaqMan murine miRNA assays and normalised to sno412 in the trachea and bronchi of 6-week-old wild-type (*n* = 9) and βENaC-Tg (*n* = 6) C57BL/6 mice. Data are represented as mean ± SEM and were compared by *t* test (*p < 0.01). All qRT-PCR experiments were performed in duplicate and included no-template controls. **(B)** Predicted binding locations of miR-221 in the full-length 5,422 bp murine ATF6 3′UTR and proposed base pair matches as predicted by TargetScan 6.2.

miR-145, miR-221 and miR-494 are conserved in mammals. The levels of these three miRNAs were measured in native airway tissues (trachea and main stem bronchi) from βENaC-transgenic C57BL/6 mice (βENaC-Tg) and wild-type littermates and normalised to SNO142. Figure 3A shows the relative expression of each miRNA in the trachea and bronchi of 6-week-old wild-type and βENaC-Tg C57BL/6 mice. In contrast to human bronchial brushings, only miR-221 was significantly increased in the airway tissues of the βENaC-Tg mice. Figure 3B depicts the full-length murine ATF6 3′UTR. Compared to the human ATF6 3′UTR which is 408 bp, the murine version is significantly longer at 5,422 bp. It contains two predicted binding locations for miR-221, but none for miR-145 or miR-494.

### miR-145, miR-221 and miR-494 target human ATF6 via repression of an ATF6 3′UTR luciferase reporter

**Figure 4:**
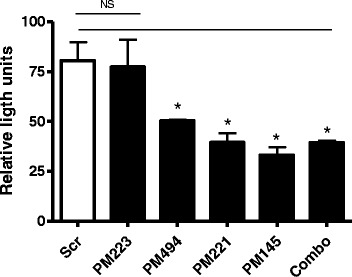
**miR**-**145**, **miR**-**221 and miR**-**494 target human ATF6.** Relative luciferase activity in HEK293 cells (1 × 10^5^ in triplicate) transiently co-transfected with full-length 408 bp human ATF6 3′UTR firefly luciferase reporter plasmid, a constitutive *Renilla* luciferase reporter plasmid (pRLSV40) and scrambled control (Scr), premiR (PM)-223 control, PM-145, PM-221 and PM-494. Firefly luciferase activity was normalised to *Renilla* luciferase activity. Transfection efficiency was monitored by visualisation of endogenous RFP expressed from the ATF6 3′UTR reporter plasmid where uniform transfection (estimated to be approximately 50%) was observed across all wells. Data are represented as mean ± SEM and were compared by *t* test (*p < 0.05). Data is representative of three experiments.

In order to determine whether human ATF6 is regulated by miR-145, miR-221 and miR-494, HEK293 cells were transiently transfected with a luciferase reporter vector containing the full-length wild-type 408 bp human ATF6 3′UTR and a reference *Renilla* luciferase reporter plasmid pRLSV40. Co-transfection with premiR (PM)-145, PM-221 or PM-494, either alone or combined, resulted in a significant decrease in luciferase gene expression from the reporter vector containing the ATF6 3′UTR compared to a scrambled control (Figure 4; *p* < 0.05). A premiR for miR-223, a miRNA not predicted to target ATF6, did not decrease luciferase activity.

### Expression of ATF6 is decreased in CF versus non-CF cell lines and bronchial brushings

**Figure 5:**
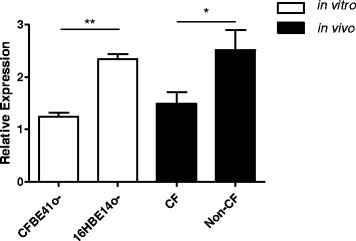
**Relative expression of ATF6 in CF versus non-** **CF bronchial epithelial cell lines and bronchial brushings.** Relative expression of ATF6 mRNA was determined by qRT-PCR and normalised to β-actin in the CF bronchial epithelial cell line CFBE41o- compared with its non-CF counterpart 16HBE14o- (three separate cultures tested in triplicate) and in bronchial brushings (CF, *n* = 8; non-CF, *n* = 9). Data are represented as mean ± SEM and were compared by *t* test (*p < 0.05, **p < 0.01). All qRT-PCR experiments for cell lines were performed in triplicate and included no-template controls.

Finally, ATF6 mRNA expression was measured in the CF versus non-CF cell lines CFBE41o- and 16HBE14o- and in a selection of CF and non-CF bronchial brushings. Figure 5 shows that expression of ATF6 mRNA, relative to β-actin mRNA, is significantly decreased in CF, both *in vitro* (*p* < 0.01) and *in vivo* (*p* < 0.05). There was no difference in ATF6 expression in homozygous versus heterozygous F508del CFTR individuals (*n* = 3 and *n* = 5, respectively).

## Discussion

ATF6 is an ER resident transcription factor involved in the unfolded protein response. Given the relatively recent discovery of miRNAs and their potential involvement in the regulation of almost all cellular processes, we examined whether miRNAs that are differentially expressed in CF airway epithelium control expression of ATF6. Here, we report decreased expression of ATF6 mRNA in F508del CF bronchial epithelium both *in vitro* and *in vivo* and correlate this observation with increased expression of miR-145, miR-221 and miR-494, three miRNAs predicted to target the ATF6 3′UTR. Of these three miRNAs, only miR-221 is predicted to regulate murine ATF6 mRNA, and in βENaC-Tg mice, expression of miR-221 is increased in native airway tissues (tracheal and bronchial tissues) compared to wild-type mice. Together, the data implicate altered miRNA expression, in particular miR-221, in controlling ATF6 levels in CF bronchial epithelium.

Based on a number of recent reports, evidence of a role for miRNA in controlling the UPR is beginning to emerge. For example, in alpha-1 antitrypsin deficiency, miR-199a-5p has been shown to control the expression of many key factors involved in UPR, including ATF6 [6]. In mice, miR-702 also regulates ATF6; however, the possible existence of miR-702 in the human genome is low [7]. Other miRNAs implicated in control of the UPR include miR-122 and miR-30c-3p [8,9]. To date, there are no reports on miRNA-mediated influences on UPR components within the CF airway epithelium.

In this study, we observed that levels of miR-145, miR-221 and miR-494 are increased in CF bronchial epithelial cells *in vitro* and *in vivo*. Previously, we reported how altered levels of miR-145 and miR-494, together with other factors, can control decreased CFTR mRNA and protein expression *in vivo* and *in vitro* [13]. Here, we extend our understanding of miR-145 and miR-494 in the context of CF bronchial epithelial cells by demonstrating their reciprocal relationship with ATF6 mRNA levels and provide evidence that miR-221 also contributes to the post-transcriptional regulation of ATF6. Regarding the expression of other miRNAs with a proven role in regulating ATF6 [6,7], neither was detected in CF and non-CF samples by *in situ* qRT-PCR arrays [10].

The βENaC-Tg mouse represents a murine model with CF-like lung disease that mimics an imbalance of CFTR-mediated anion secretion and ENaC-mediated Na^+^ absorption characteristic of human CF airways. This imbalance causes airway surface dehydration and a spontaneous lung disease phenotype that shares key features with human CF including mucus plugging, chronic airway inflammation and reduced clearance of bacteria [3,4]. Although the three miRNAs under investigation here are conserved between humans and mice, only miR-221 was found to be similarly increased in the trachea and bronchi of 6-week-old βENaC-Tg versus wild-type mice. Interestingly, bioinformatic analysis of the murine ATF6 3′UTR revealed two predicted miRNA recognition elements for miR-221, whereas miR-145 and miR-494 were not identified as potential regulators. Thus, βENaC-Tg mice may represent a useful model for further studies into the biology and function of miR-221 in CF lung disease. Regarding the age of the mice, 6-week-old mice are comparable to young adults as previously published for the βENaC-overexpressing mouse and other models of experimental lung disease in mice [14,15], and thus similar to the age of the CF patients studied here. Whether miRNA expression patterns differ in paediatric versus adult CF patients has not been studied to date.

More than 95% of the misfolded protein encoded by the F508del-CFTR mutation is retained in the ER and subsequently degraded by the proteasome [16]; consequently, less CFTR reaches the apical membrane and ion transport is impaired. Conflicting opinions exist as to whether the aberrant folding of F508del-CFTR elicits an ER stress response and consequently activates the UPR. A number of studies suggest this is the case [17,18], whilst others propose that ER stress arises as a direct result of inherent inflammatory factors and chronic infection [19-21]. Therefore, it remains controversial whether mutant CFTR itself causes ER stress, UPR induction and subsequent inflammation or whether the chronic inflammation observed in CF is the primary ER stress effector leading to UPR activation independent of the aberrantly folded CFTR. A number of studies have reported a lack of UPR activation in F508del-CFTR homozygous primary tracheal cell cultures [22] and nasal epithelial CF15 cell lines [23] under basal conditions. Consistent with this, increased ER density and Ca^2+^ signals observed in short-term primary cultures of F508del-CFTR homozygous CF cells which disappear over time (30 to 40 days) can be reproduced in normal bronchial epithelial cells stimulated with supernatant from CF lung mucopurulent material. This suggests that airway infection and inflammation and not the presence of mutant CFTR instigate an ER stress response [20,21]. Our data supports these studies. We observed lower levels of ATF6 mRNA in CF versus non-CF cells under basal conditions.

There are a number of limitations associated with this study. For example, although inhibition of luciferase activity in a vector containing the full-length human ATF6 3′UTR indicated that each of the three miRNAs were capable of decreasing luciferase expression, in order to demonstrate that miR-145, miR-221 and miR-494 directly target the human ATF6 3′UTR, additional experiments using reporter constructs in which the predicted MREs for the individual miRNAs are deleted or mutated would be required. Unfortunately, this was not possible at this time. Also, whilst it would be ideal to demonstrate decreased ATF6 protein levels *in vivo*, bronchial brushings are clinical samples obtained from a highly invasive procedure that yields relatively few cells. The priority in this study was to assess miRNA and mRNA expression levels in these rare samples, thus precluding their use for Western blot analysis. Finally, miRNA and ATF6 mRNA expressions were examined under basal rather than agonist-induced ER stress conditions. This approach was taken in order to establish the behaviour and relationship of these factors in the context of CF epithelial cells; future work should investigate how infection or inflammation may impact on the fundamental processes described here.

## Conclusion

Taken together, the data here demonstrate a role for miR-145, miR-221 and miR-494 in regulating ATF6. The observation that miR-221 is increased in CF versus non-CF bronchial brushings and also in native airway tissues of βENaC-Tg versus wild-type mice indicates that this miRNA is both structurally and functionally conserved between humans and mice. Various miRNA-targeting therapeutics are under investigation for cystic fibrosis, and it has been demonstrated in proof-of-concept studies that it is feasible to deliver miRNA modulators into primary and transformed CF nasal and bronchial epithelial cells in culture [24-26]. In the future, manipulation of miR-221 levels *in vivo* using miRNA overexpression strategies could be used to decrease levels of ATF6 in CF, thereby limiting ER stress-mediated inflammation. This strategy may have therapeutic potential for CF or other conditions where the UPR plays a role.

## Abbreviations

ATF6: activating transcription factor 6
CF: cystic fibrosis
CFTR: CF transmembrane conductance regulator
ER: endoplasmic reticulum
miRNA: microRNA
MRE: miRNA recognition element
PM: premiR
PWCF: people with CF
RFP: red fluorescent protein
UPR: unfolded protein response
WT: wild-type
